# PARP inhibitor olaparib enhances the efficacy of radiotherapy on *XRCC2*-deficient colorectal cancer cells

**DOI:** 10.1038/s41419-022-04967-7

**Published:** 2022-05-28

**Authors:** Changjiang Qin, Zhiyu Ji, Ertao Zhai, Kaiwu Xu, Yijie Zhang, Quanying Li, Hong Jing, Xiaoliang Wang, Xinming Song

**Affiliations:** 1 Department of Gastrointestinal Surgery, Huaihe Hospital of Henan University, Kaifeng, China; 2 Department of Gastrointestinal and Pancreatic Surgery, The First Affiliated Hospital of Sun Yat-sen University, Guangzhou, China; 3 Department of Medical Oncology, Huaihe Hospital of Hennan University, Kaifeng, China; 4 Department of Pathology, Huaihe Hospital of Hennan University, Kaifeng, China; 5 Department of General Surgery, Qingpu Branch of Zhongshan Hospital Affiliated to Fudan University, Shanghai, China

**Keywords:** Colorectal cancer, Prognostic markers

## Abstract

The use of PARP inhibitors in combination with radiotherapy is a promising strategy to locally enhance DNA damage in tumors. Loss of XRCC2 compromises DNA damage repairs, and induced DNA damage burdens may increase the reliance on PARP-dependent DNA repairs of cancer cells to render cell susceptibility to PARP inhibitor therapy. Here we tested the hypothesis that XRCC2 loss sensitizes colorectal cancer (CRC) to PARP inhibitor in combination with radiotherapy (RT). We show that high levels of XRCC2 or PARP1 in LARC patients were significantly associated with poor overall survival (OS). Co-expression analyses found that low levels of PARP1 and XRCC2 were associated with better OS. Our in vitro experiments indicated that olaparib+IR led to reduced clonogenic survival, more DNA damage, and longer durations of cell cycle arrest and senescence in XRCC2-deficient cells relative to wild-type cells. Furthermore, our mouse xenograft experiments indicated that RT + olaparib had greater anti-tumor effects and led to long-term remission in mice with *XRCC2*-deficient tumors. These findings suggest that XRCC2-deficient CRC acquires high sensitivity to PARP inhibition after IR treatment and supports the clinical development for the use of olaparib as a radiosensitizer for treatment of XRCC2-deficient CRC.

## Introduction

Colorectal cancer (CRC) is among the most common cancers and a leading cause of cancer deaths worldwide [1]. The National Comprehensive Cancer Network guidelines currently recommend neoadjuvant chemoradiotherapy (neoCRT) as standard treatment for locally advanced rectal cancer (LARC) because it can significantly increase local control and improve cancer-specific survival [2, 3]. Most LARC patients who receive neoRT/neoCRT experience some tumor response, but other patients are resistant to this therapy. Many recent investigations reported that the response of an individual CRC patient is related to specific gene expression patterns in cancer cells [4]. The X-ray repair complementing defective repair in Chinese hamster cells 2 gene (*XRCC2*) codes for a DNA repair protein, and XRCC2 expression in some cancers is associated with increased radioresistance. For example, a previous study showed that the 72% of LARC patients who tested negative for XRCC2 expression in cancer tissues had good pathologic responses and prognoses following neoRT; however, the other 28% of patients who tested negative for XRCC2 developed radioresistance and had poor prognoses [5]. It seems possible that when cancer cells lose XRCC2 function, they develop an alternative or compensatory pathway(s) for DNA repair. Therefore, agents that specifically target and radiosensitize tumor cells may be an effective treatment for patients with radioresistant tumors. For example, tumor cells that have BRCA inactivation remain sensitive to inhibition of poly (ADP-ribose) polymerase (PARP) due to their deficiency in homologous recombination repair (HRR) [6, 7].

RT kills cancer cells mostly by inducing DNA damage, and double-strand breaks (DSB) are the most toxic type of DNA damage [8]. Cells repair DSBs by non-homologous end joining (NHEJ) or HRR [9]. The PARP family of proteins plays a key role in a variety of cellular processes that includes DNA repair, chromatin modulation, and aspects of the replication stress response [10, 11]. PARP1 is the first and best-characterized member of the PARP family. PARP1 functions in the detection and initiation of DNA repair, and plays a role in repairing most types of DNA damage, including single-strand breaks (SSBs) and DSBs [12]. Because of the essential role of PARP in the recognition and repair of DSBs, several researchers examined the effect of PARP inhibitors on the sensitization of tumors defective in HRR following induction of DSBs with ionizing radiation (IR). They reported the accumulation of DNA DSBs and increased cell death [13–15].

HRR activity in tumor cells is a key factor for predicting whether treatment with a PARP1 inhibitor will be successful [16]. The XRCC2 protein functions in HRR, in that it participates in the repair of DSBs [17, 18]. Previous research showed that inhibition of XRCC2 sensitized CRC cells to radiation due to the inhibition of HRR [5]. This led us to hypothesize that PARP1 inhibitors have the potential for use as radiosensitizers in patients with XRCC2-deficient CRC because they increase the therapeutic benefit provided by RT. Therefore, we examined LARC patients, in vitro models of CRC, and an in vivo model of CRC in which the tumors had different XRCC2 status to assess the radiosensitizing effect of olaparib, a drug approved for the treatment of several cancers with BRCA mutations.

## Materials and methods

### Patients

A total of 167 LARC patients were examined at Sun Yat-sen University and Henan University. All patients had newly diagnosed LARC and received neoCRT from January 2010 to December 2016. The inclusion criteria were the presence of one primary lesion, completion of a standard neoCRT regimen, receipt of radical surgical resection, and completion of adjuvant chemotherapy with a capecitabine, XELOX, or mFOLFOX6 regimen. All biopsy samples were collected before neoCRT, as previously described [5]. The use of tissue blocks was approved by the Institutional Ethics Review Board of Sun Yat-sen University and Henan University. All patients provided written informed consent for participation.

### CRC cell lines

Two human CRC cell lines (HCT116 and SW480) were purchased from American Type Culture Collection (ATCC, Manassas, VA, USA) and were grown according to the guidelines of the ATCC. Both cell lines were verified by short tandem repeat analysis (China Center for Type Culture Collection, Wuhan, China). The cells were cultured in DMEM (DMEM, Biological Industries, USA) supplemented with 10% Fetal Bovine Serum (FBS, Hyclone, USA) and 1% penicillin/streptomycin. All cells were cultured in a humidified air incubator containing 5% CO_2_ at 37 °C. Cells with stable knockdown of *XRCC2* (Sh-XRCC2) or with nonsilencing vector (control) were generated as previously described [5, 19]. Quantitative RT-PCR and western blotting were used to determine the extent of knockdown.

### Western blotting

For western blotting, proteins were first separated using 8–10% SDS-PAGE and the proteins were then electrotransferred to PVDF membranes (Millipore, Billerica, MA, USA). Then the membranes were blocked with 5% bovine serum albumin (BSA) for 1 h (Beyotime, Beijing, China) in TBS-T, incubated with specific primary antibodies overnight at 4 °C, and then incubated with a rabbit or mouse horseradish peroxidase-coupled secondary antibody for 1 h. Antibody binding was determined using enhanced chemiluminescence (Millipore, Billerica, MA, USA).

### Quantitative real-time PCR

Total RNA was extracted and qRT-PCR was performed as described previously [5, 19]. All experiments were performed at least three times.

### Immunofluorescence

Cells were transferred into confocal dishes. Then 4% paraformaldehyde was used for fixation, 0.5% Triton X-100 was used for permeabilization, and 3% BSA was used for blocking. Anti-γH2AX (1:50, D17A3, Cell Signaling Technology [CST], Danvers, MA, USA) antibodies were added at 4 °C overnight. Then, the sample was incubated with DyLight 488 AffiniPure Goat Anti-rabbit IgG (1:200, Abbkine, Redlands, CA, USA) for 1 h in the dark at room temperature, and 4’,6-diamidino-2-phenylindole (DAPI) was used for costaining. Cells were examined using confocal laser scanning microscopy (Zeiss, Germany) and the number of foci of γH2AX (marker of DSB) per nucleus was determined. An average of 100 nuclei per sample were examined.

### Clonogenic cell survival assay

The clonogenic survival assay was performed as described in detail previously [5]. Briefly, 200 transfected SW480 or HCT116 cells were seeded in 6-well plates (35-mm diameter well). After the addition of vehicle (dimethylsulfoxide [DMSO]) or olaparib for 6 h, a varying dose of IR was applied (0–8 Gy), and the cells were then maintained in an incubator at 37 °C with 5% CO_2_ for 10 to 14 days. Then methanol was used for fixation, crystal violet was used for staining, and colonies with >50 cells were counted. All experiments were performed in triplicate and repeated three times.

### Flow cytometry

Cell cycle analysis of cultured cells was determined by harvesting at 72 h after treatment, fixation overnight with 70% ethanol at −20 °C, and staining with 20 mg/mL propidium iodide (PI) in a buffer (1% Triton X-100 and 100 mg/mL RNase A) for 30 min. The FACSCalibur unit (Becton Dickinson, Franklin Lakes, NJ, USA) with the ModFit LT version 2.0 software was used to determine the levels of DNA. All experiments were repeated at least three times.

### Senescence-associated β-galactosidase assay

A kit for staining senescent cells using β-galactosidase (β-gal) was purchased from CST (#9860), and cells were fixed and stained according to the manufacturer’s protocol. For in vivo experiments, frozen sections of tumor xenografts (5-μm thick) were added to 2% glutaraldehyde for fixation, and then staining was performed as described in the in vitro experiments (above). Five images of randomly selected fields were recorded using an inverted microscope. Then blue-stained (senescent) cells and unstained (non-senescent) cells were counted using a computer. The percentages of positive cells were plotted using GraphPad Prism version 8.0.

### Immunohistochemistry

IHC assays were performed as previously described in detail [20]. First, formalin-fixed and paraffin-embedded tissue sections (4-μm thick) were prepared. The sections were then deparaffinized and rehydrated, antigen retrieval and endogenous peroxidase inactivation were performed, and blocking was applied. All slides were incubated overnight at 4 °C with anti-XRCC2 (1:200, #ab180752; Abcam), anti-PARP1 (1:200; #ab32138; Abcam), or anti-γH2AX (1:100; D17A3; Cell Signaling Technology), and then with a secondary antibody (Vectastain ABC kit). Finally, 3, 3-diaminobenzidine (DAB) was used for staining and hematoxylin for counter-staining.

### Animals and in vivo studies

The Henan University Animal Care and Use Committee approved all in vivo experiments. Mice were sacrificed by an inhalant overdose of carbon dioxide (CO_2_, 10–30%), followed by cervical dislocation. All efforts were made to minimize suffering. Experiments using tumor xenografts were performed as previously described in detail [21]. First, 6 × 10^6^ SW480 cells were suspended in 100 μL of PBS. Cells were then subcutaneously implanted into the right flanks of female BALB/c nude mice that were 4–5 weeks old and weighed 15–18 g (SLRC Laboratory Animal Co). When the tumor volume was about 100 mm^3^, mice were separated into a DMSO group, olaparib group, RT + DMSO group, or RT + olaparib group. Mice received these treatments by oral gavage once per day for 12 consecutive days. Mice in the RT + DMSO and RT + olaparib groups received fractionated radiotherapy (2 Gy every other day for 5 days) at 1 h after oral gavage; a lead plate was used to provide coverage and assure that IR was only applied to the xenograft region. Tumors were measured using a digital caliper and volume was calculated as 0.52 × width^2^ × length. Mice were euthanized and tumors were harvested 2 days after the last dose of olaparib or oralipib+RT. For long-term studies, mice received oralipib+RT therapy and were monitored for tumor growth until 30 days after RT. Mice were sacrificed by an inhalant overdose of carbon dioxide (CO_2_, 10–30%), followed by cervical dislocation. All efforts were made to minimize suffering.

### Statistical analysis

All results were confirmed in at least three independent experiments; one-way and two-way analysis of variance tests were used for comparisons of the results of the in vitro and in vivo experiments, and correction for multiple comparisons was performed using the Tukey or Sidak test, as appropriate. Each value was expressed as mean ± standard deviation (SD). A *P* value <0.05 was considered significant. All statistical analyses were performed using GraphPad Prism version 8.0.

## Results

### High expression of XRCC2 and PARP1 is associated with poor prognosis in LARC patients who received neoRT

Previous research showed that *XRCC2* expression in pretreatment biopsy specimens was associated with response to neoRT in LARC patients [5], and that greater expression of *XRCC2* was associated with reduced sensitivity to PARP1 inhibition [22]. We therefore investigated the relationship of XRCC2/PARP1 coexpression with the efficacy of RT in patients with LARC.

**Fig. 1:**
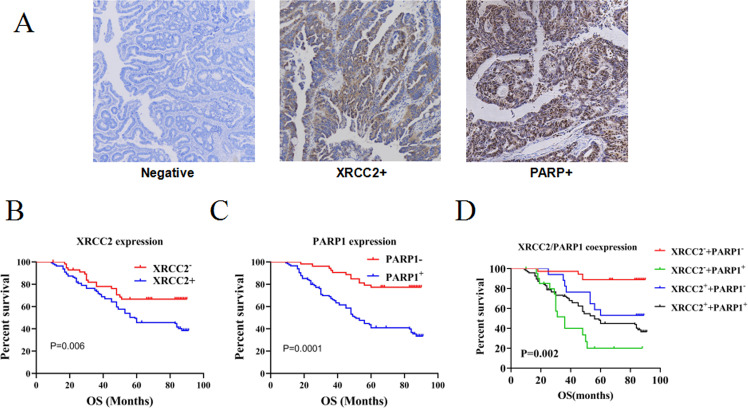
Prognostic significance of XRCC2 and PARP1 expression in LARC patients who received neoRT. **A** Representative immunohistochemical images showing the expression of XRCC2 and PARP1 in rectal tissues (×200). **B** Kaplan–Meier analysis of the effect of XRCC2 expression on OS. **C** Kaplan–Meier analysis of the effect of PARP1 expression on OS. **D** Kaplan–Meier analysis of the effect of XRCC2/PARP1 co-expression on OS.

The expression of XRCC2 and PARP1 in biopsy tumor specimens collected from 167 LARC patients before treatment with neoCRT followed by surgery indicated that 57 samples (34.1%) were XRCC2^+^ and 52 samples (31.1%) were PARP1^+^ (Fig. 1A). Kaplan–Meier survival analysis (Fig. 1B, C) indicated poor overall survival (OS) in patients with high levels of XRCC2 (*P* = 0.006) or PARP1 (*P* = 0.0001). XRCC2/PARP1 coexpression analysis in the whole cohort indicated that patients who had tumors with low coexpression had better OS (*P* = 0.002; Fig. 1D). These data suggest that simultaneous targeting of XRCC2 and PARP1 could be a promising treatment for selected LARC patients. We therefore further examined the potential of this treatment regimen using in vitro and animal studies.

### Olaparib increased the radiosensitivity of XRCC2-deficient CRC cells

**Fig. 2:**
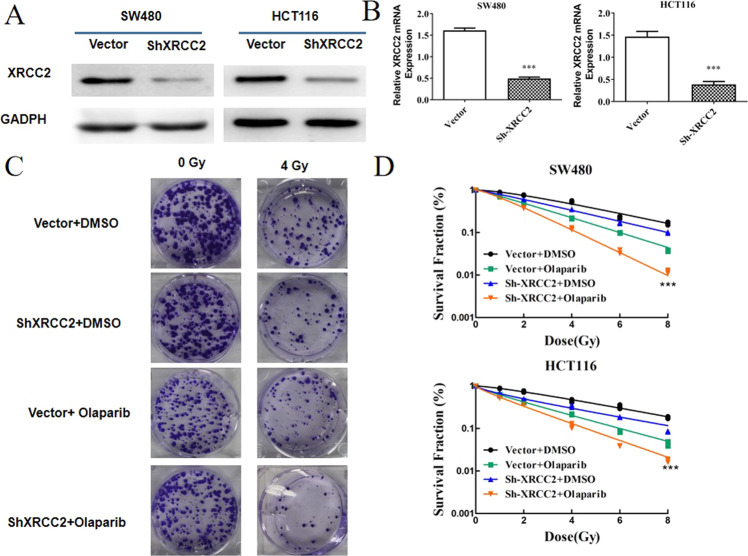
Effect of olaparib on the radiosensitivity of XRCC2-deficient rectal cancer cells. **A** Western blotting of XRCC2 in SW480 and HCT116 cells with (shXRCC2) and without (vector) *XRCC2* knockdown. **B** qRT-PCR of *XRCC2* in SW480 and HCT116 cells with (shXRCC2) and without (vector) *XRCC2* knockdown. ***P < 0.001. **C** Representative images of the clonogenic cell survival assays in the different treatment groups. **D** Effect of IR dose on survival of cells in different groups. Lines are from statistical fits to the mean values from three independent experiments to a linear-quadratic (multi-target/single-hit) model.

Previous studies established a line of CRC cells with successful knockdown of *XRCC2* [5, 19]. We first confirmed the knockdown of XRCC2 in these cells using western blotting (Fig. 2A) and qRT-PCR (Fig. 2B). We then examined the survival of these cells following combined treatment with olaparib (a PARP inhibitor) and IR using the clonogenic survival assay (Fig. 2C, D). The results indicated the olaparib+IR regimen significantly reduced survival relative to IR alone (all *P* < 0.05), indicating that olaparib radiosensitized these CRC cells. Importantly, *XRCC2*-deficient cells had greater sensitivity to IR and greater olaparib-mediated radiosensitization than cells with empty vectors.

### Olaparib+IR increased DNA damage in XRCC2-deficient CRC cells

**Fig. 3:**
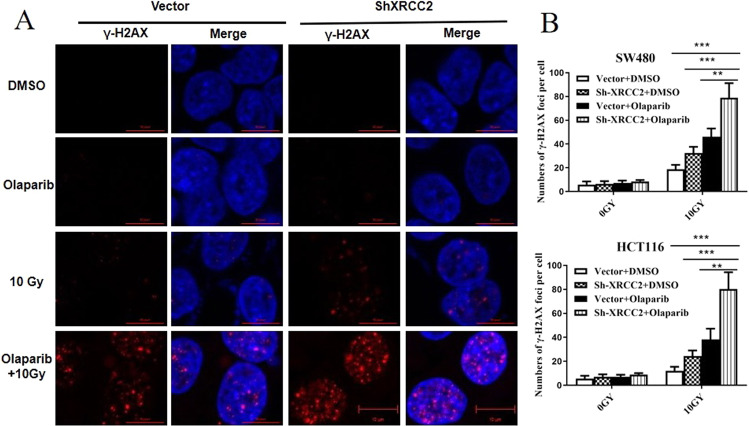
Effect of olaparib on the persistence of radiation-induced double-strand breaks in *XRCC2*-deficient rectal cancer cells. **A** Representative immunofluorescence images of γ-H2AX foci in SW480 cells at 48 h in the different treatment groups (×400). **B** Average number of γ-H2AX foci per nucleus in the different treatment groups. Results show the means and standard errors of the means (SEMs) from three independent experiments. **P < 0.01, ***P < 0.01.

IR causes DSBs and induces the rapid production of γH2AX, a marker of impaired cellular capacity to repair DSBs [23, 24]. We thus investigated the effects of olaparib on IR-induced DNA damage by using immunofluorescence to measure γH2AX in *XRCC2*-deficient CRC cells. After 48 h, treatment with olaparib alone led to no significant DNA damage, but treatment with olaparib+IR led to increased levels of γH2AX (Fig. 3A, B). Importantly, irradiated XRCC2-deficient cells had more γH2AX foci than XRCC2-expressing cells at 48 h when treated with olaparib. These data suggest that treatment with olaparib+IR led to more DNA damage in *XRCC2*-deficient CRC cells than in control cells with empty vectors.

### Olaparib promoted G2/M phase arrest in XRCC2-deficient CRC cells after RT

**Fig. 4:**
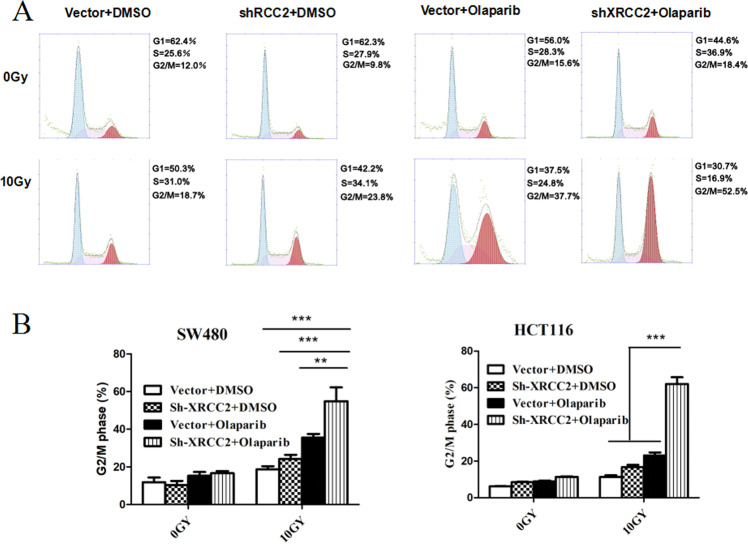
Effect of olaparib on sensitization of ***XRCC2***-deficient cancer cells to IR-induced phase G2/M arrest. **A** Cell cycle profiles of cells with (sh-XRCC2) and without (vector) *XCRCC2* knockdown at 48 h after the indicated treatment. **B** Quantification of flow cytometry results in the different treatment groups. Results show the means ± SDs from three independent experiments. **P < 0.01, ***P < 0.001.

Sustained arrest at the G2/M growth phase is a well-established cellular response to DNA damage [25]. Thus, we examined the effect of olaparib+IR on the fraction of *XRCC2*-deficient CRC cells that were arrested in the G2/M phase. In particular, we performed FACS analysis to compare CRC cells that had empty vectors with CRC cells that had *XRCC2* depletion that were treated with olaparib, IR, or both (Fig. 4). The results showed that treatment with olaparib had no effect on the number of *XRCC2*-deficient cells in phase G2/M. However, IR + olaparib led to a significant arrest of the cell cycle at the G2/M phase in both cell lines (*P* < 0.01; Fig. 3A, B). Furthermore, olaparib+IR increased the percentage of cells in phase G2/M in *XRCC2*-deficient CRC cells compared to CRC cells with empty vectors (*P* < 0.01; Fig. 4A, B).

### Olaparib accelerated senescence when combined with IR in XRCC2-deficient cells

**Fig. 5:**
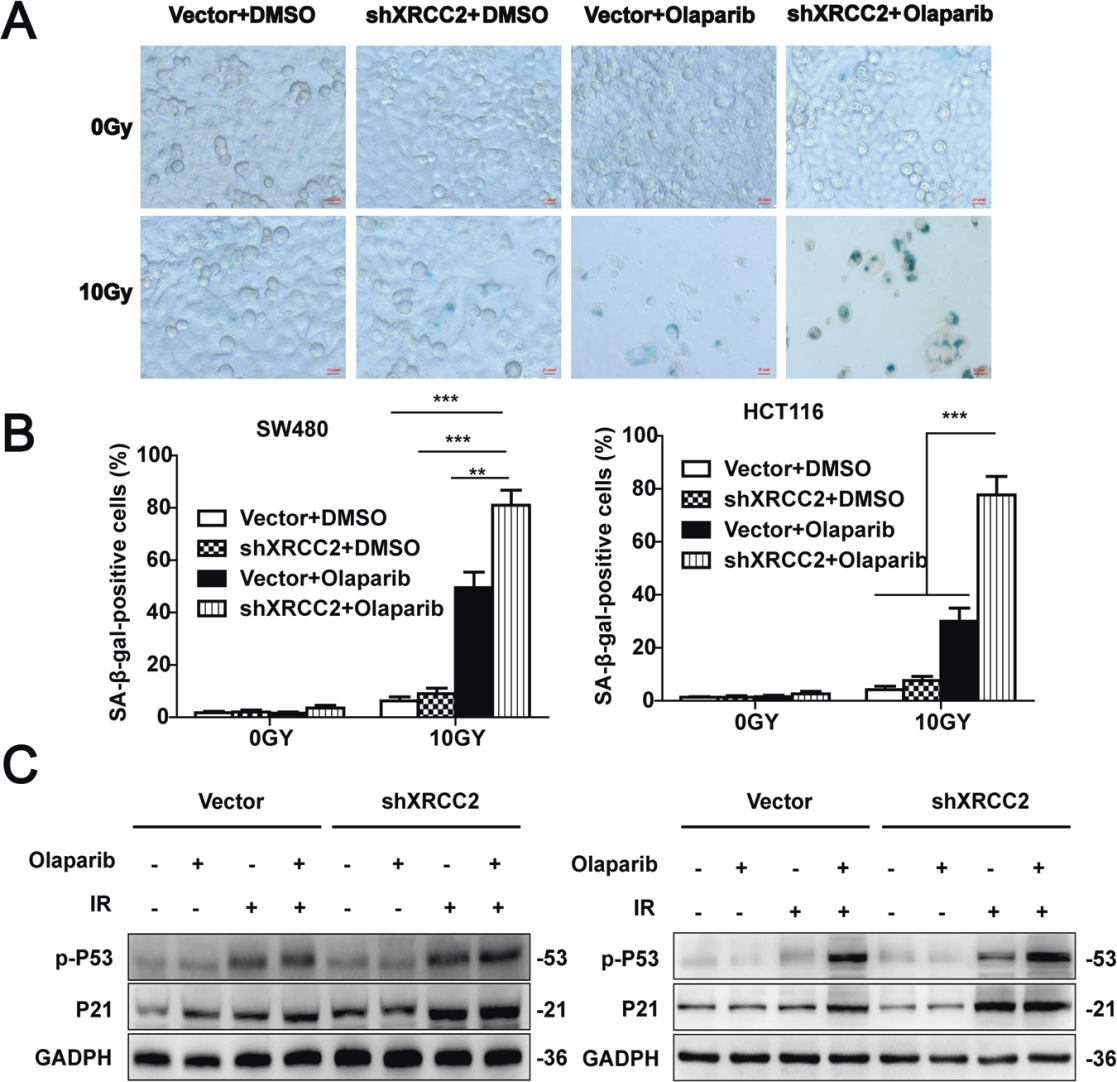
Effect of olaparib on senescence after in vitro IR treatment. **A** Representative images of senescence (β-gal activity) after 48 h in the different treatment groups (×40). **B** Quantification of microscopy results. Results show means ± SDs from three independent experiments. **P < 0.01, ***P < 0.001. **C** Representative western blotting of phospo-P53 and P21 in SW480 cells (left) and HCT116 cells (right) in the different treatment groups.

Irreversible cell cycle arrest is a key feature of accelerated senescence. Thus, we analyzed the effect of different treatments on cell senescence using the β-gal assay (Fig. 5A). IR + olaparib significantly increased the proportion of β-gal-positivity in *XRCC2*-deficient cells relative to cells with empty vectors (*P* < 0.001; Fig. 5B). Agents that damage DNA primarily promote senescence *via* the p53/p21 signaling pathway [26], so we also analyzed the effects of different treatments on the levels of phospho-p53 and p21. The results indicated that *XRCC2*-deficient cells and cells with empty vectors had increased levels of phospho-p53 and p21 at 48 h after 10 Gy of IR, and olaparib treatment increased this effect. Moreover, the olaparib+IR treatment led to higher levels of phospho-p53 and p21 in *XRCC2*-deficient cells than in cells with empty vectors (Fig. 5C). These results indicated that olaparib enhanced the effect of IR in the induction of cell senescence.

### Olaparib increased the radiosensitivity of XRCC2-deficient cells in a mouse xenograft model

**Fig. 6:**
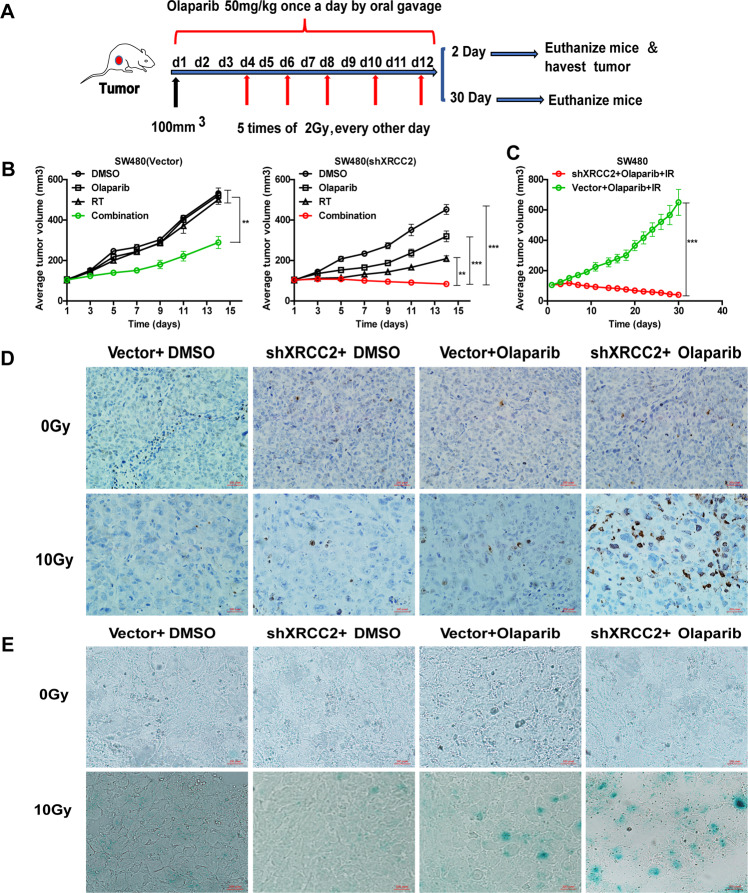
Effect of olaparib and IR on growth and senescence of *XRCC2*-deficient tumor xenografts in mice. **A** Experimental design. **B** Change of tumor volume over 2 weeks in the different groups that received SW480 cells with empty vectors (left) or shXRCC2 (right). Data are presented as means ± SEMs (*n* = 5); **P < 0.01, ***P < 0.001. **C** Change of tumor volume over 4 weeks in the two indicated groups. Data are presented as means ± SEMs (*n* = 5); ***P < 0.001. **D**, **E** Representative immunohistochemical staining of γ-H2AX (top) and β-gal activity (bottom) in the different groups from tumors harvested 2 days after treatment with Olaparib with or without IR (×40).

We evaluated the effect of IR on olaparib sensitivity using mice xenograft experiments in which the two SW480 cell lines only differed in *XRCC2* status (Fig. 6A). Strikingly, olaparib+RT prevented the growth of *XRCC2*-deficient tumors and was more effective than either treatment alone (Fig. 6B). We also determined whether olparib+RT led to long-lasting effects by following mice that received this therapy for 30 days (2 weeks after the final treatment). The results indicated that *XRCC2*+/+ tumors regrew quickly following treatment cessation, but sh*XRCC2* tumors continued to shrink (Fig. 6C).

We then assessed the broader implications of these findings by harvesting tumors from all mice 2 days after the last treatment (Fig. 6A). The results indicated that γH2AX expression of tumor tissues following olaparib+RT treatment was significantly greater in *XRCC2*-deficient CRC cells (Fig. 6D). Although there was negligible staining following treatment with olaparib alone, there was notably increased activity of β-gal following treatment with olaparib+RT, especially in *XRCC2*-deficient CRC cells (Fig. 6E). The results of these xenograft tumor model experiments confirmed that olaparib increased the radiosensitivity of *XRCC2*-deficient CRC cells.

## Discussion

PARP1 inhibitors are therapeutic agents that increase cell death in tumors with deficiencies in HRR-mediated DNA repair, such as those with BRCA1 mutations [10, 27]. Thus, CRC cells that respond to PARP1 inhibitors are likely to have defects in DSB repair enzymes, even though BRCA mutations are rare in patients with CRC [28]. Previous studies showed that *XRCC2*-deficient CRC cells were sensitive to 5-fluorouracil (5FU) and RT [5, 19]. In particular, a study of 67 LARC patients treated with neoRT between 2010 and 2012 indicated that XRCC2 overexpression was associated with RT resistance and poor OS [5]. We examined these 67 patients and an additional 100 patients with LARC who were treated from 2013 to 2016 and described the results of all 167 patients herein. Our analysis of these 167 patients confirmed the previous observations. We also found that PARP1 overexpression was negatively associated with OS, and co-expression analyses indicated that low co-expression of PARP1 and XRCC2 was associated with a better OS. These results thus provide evidence that a PARP1 inhibitor (olaparib) may enhance the effect of RT on *XRCC2*-deficient CRCs.

Death of human tumor cells following RT is caused mainly due to the induction of DSBs, damage that can be repaired by HRR or NHEJ [29]. A recent study showed that XRCC2 deficiency sensitized CRCs to RT [5]. However, not all XRCC2-deficient cancer cells were sensitive to IR in vitro [5, 29]. IR induces complex DNA lesions that require repair *via* the HRR pathway (which depends on XRCC2), although the NHEJ pathway (which does not require XRCC2) can repair most of these lesions [29, 30]. This might explain the differences in radiosensitivity in our HRR deficient model.

Radiosensitization by treatment with a PARP1 inhibitor is apparently due to the inhibition of base excision repair. This leads to a delay in the repair of SSBs, and the collision of these SSBs with replication forks transforms them into 1-ended DSBs. These DSBs can only be repaired by HRR [31]. Thus, the application of a PARP1 inhibitor with RT leads to greater inhibition in BRCA2− than BRCA2+ breast cancers [32]. Other studies reported that radiosensitization by PARP1 inhibitors was greater when a variety of DNA DSB repair deficiencies were present, such as deficiencies in Ligase IV and RAD51C [33, 34]. Our results are consistent with these previous studies, in that olaparib treatment led to radiosensitization of CRC cells, particularly in tumors with XRCC2 deficiencies. Our in vitro studies of the effect of IR + olaparib indicated radiosensitization in XRCC2-deficient cell lines, manifested as decreased colony formation, increased γ-H2AX persistence, increased cellular senescence, and cell cycle arrest. We also confirmed the effectiveness of this combination treatment in vivo using XRCC2-deficient tumor xenografts, which had delayed growth relative to xenografts with empty vectors.

We also compared the extent of DNA breaks from IR alone *vs*. IR + olaparib by measuring the levels of γ-H2AX. As expected, the different responses to these treatments were associated with different residual levels of total DNA damage. This may be because there is a need for repeated cycles of DNA synthesis so that unrepaired single-strand DNA breaks caused by IR are converted to DSBs, which cannot be repaired because of XRCC2 deficiency. Cell cycle regulation is an important biological process that also affected radiosensitivity, and cells are typically most sensitive during the G2/M phase [35]. There is evidence that PARP may promote G2/M arrest following genotoxic stress [36]. Our data on different CRC cell lines showed that IR + olaparib treatment significantly increased the proportion of cells in the G2/M phase compared to IR alone and olaparib alone. Moreover, our results indicated a significant increase in XRCC2-deficient CRC cells after the combination treatment.

The promotion of cellular senescence can increase the effectiveness of RT [37]. A previous study of the effect of a PARP1 inhibitor combined with RT showed that radiosensitization manifested predominantly as an extension of growth arrest and senescence and that there was little or no contribution from apoptosis [38]. Our present results showed that IR + olaparib treatment led to significantly increased cellular senescence compared to either treatment alone, and the greatest senescence was in XRCC2-deficient cells. These results are in accordance with the study of Alotaibi et al., who demonstrated that PARP inhibitors did not increase radiation-induced apoptosis in DNA repair-deficient tumor cells, but did markedly enhance growth arrest and senescence [33]. This lack of a role for apoptosis is not surprising, because RT is believed to mainly kill cells by induction of “mitotic catastrophe” (aberrant mitosis), in which large non-viable cells that have micronuclei or multiple nuclei are formed [39, 40].

Phospho-p53 activates many cellular responses, including initiation of senescence and cell cycle arrest after IR [41, 42]. The results presented here showed that olaparib+IR increased the level of phospho-p53 in XRCC2-deficient CRC cells. We also observed increased p21 levels in XRCC2-deficient CRC cells after IR, which is noteworthy because p21 inhibits cyclin-dependent-kinases, phospho-p53 induction of p21 leads to arrest at the G2/M phase. We showed that IR treatment led to a significant arrest of the cell cycle in phase G2 and that this arrest persisted for 72 h. In addition to its role in cell cycle arrest, p21 activation is also an initial step of accelerated senescence [43].

## Supplementary information

Original Data

## Data Availability

The data that support the findings of this study are available from the corresponding author upon reasonable request.
