# Supplementary material for: PARP inhibitor olaparib enhances the efficacy of radiotherapy on XRCC2-deficient colorectal cancer cells
Source: Cell Death Dis. 2022 May 28;13(5):505. doi: 10.1038/s41419-022-04967-7 (PMC9148313; doi:10.1038/s41419-022-04967-7)

Original Data for

PARP Inhibitor Olaparib Enhances the Efficacy of Radiotherapy on XRCC2-deficient Colorectal Cancer Cells

Changjiang Qin, Zhiyu Ji, Ertao Zhai, Kaiwu Xu, Yijie Zhang ,Quanying Li, Hong Jing, Xiaoliang Wang , Xinming Song

Original WB images

Figure 2A

XRCC2

GADPH

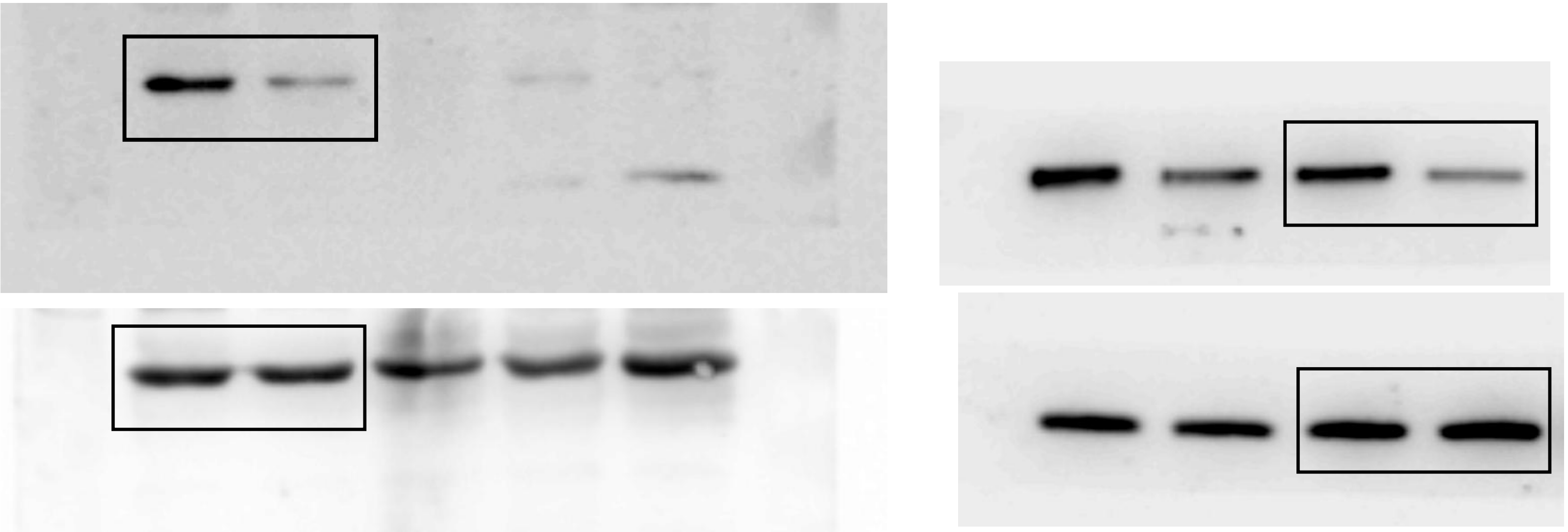

Figure 5C

p-P53

P21

GADPH

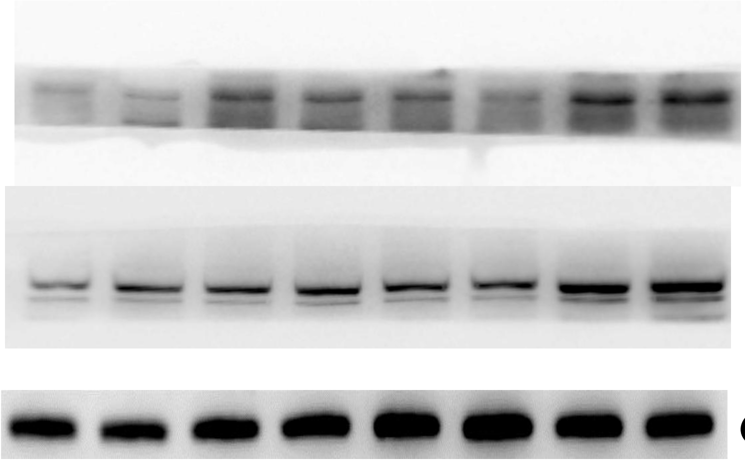

p-P53

P21

GADPH

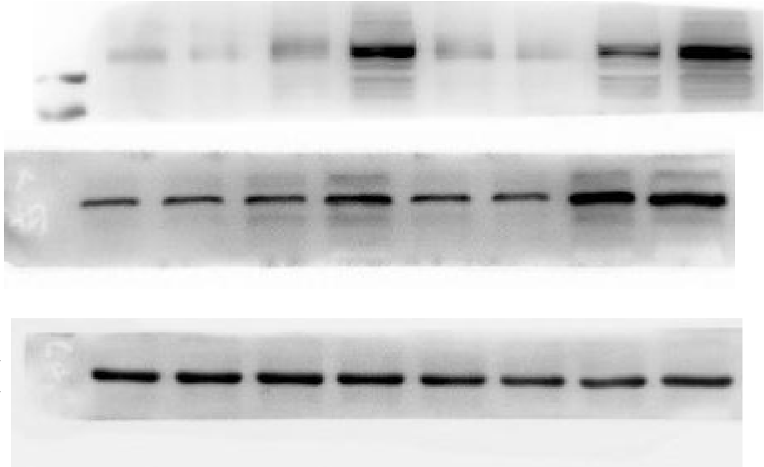

Supplement: Supplementary file 1 — Original Data [file 41419_2022_4967_MOESM1_ESM.pdf]
